# Three novel sequencing types from seventeen *Staphylococcus aureus* genomes isolated from dairy cows milk in the Free State Province of South Africa

**DOI:** 10.1128/MRA.00739-23

**Published:** 2023-11-15

**Authors:** Ntelekwane George Khasapane, Stanford Kwenda, Zamantungwa Thobeka Happiness Khumalo, Sebolelo Jane Nkhebenyane, Oriel Thekisoe

**Affiliations:** 1 Department of Life Sciences, Centre for Applied Food Sustainability and Biotechnology, Central University of Technology, Bloemfontein, South Africa; 2 Sequencing Core Facility, National Institute for Communicable Diseases, National Health Laboratory Service, Johannesburg, South Africa; 3 Clinvet International, Study Operations, Uitsig Road, Universitas, Bloemfontein, South Africa; 4 Department of Veterinary Tropical Diseases, Faculty of Veterinary Science, University of Pretoria, Onderstepoort, South Africa; 5 Unit for Environmental Sciences and Management, North-West University, Potchefstroom, South Africa; Loyola University Chicago, Chicago, Illinois, USA

**Keywords:** whole genome sequencing, bovine, subclinical mastitis, *Staphylococcus aureus*, sequencing types

## Abstract

*Staphylococcus aureus* is one of the major pathogens causing bovine mastitis, which results in huge economic losses in the dairy industry worldwide. Here, we report genome sequences of 17 *S*. *aureus* strains, with three novel sequencing types (ST8495, ST8500, and ST8501) isolated from the milk of dairy cows with subclinical mastitis.

## ANNOUNCEMENT

Bovine mastitis is an inflammatory condition that has several causes, which can be pathogenic or environmental (1, 2). *Staphylococcus aureu*s is a bacterium that is usually linked to subclinical mastitis (3). Additionally, *S. aureus* isolates from cattle are a major contributor to foodborne illnesses (4–6). The current announcement reports on 17 *S. aureus* genome sequences with three novel sequencing types isolated from milk of cows with bovine subclinical mastitis from three municipalities, namely, Maluti-A-Phofung (28°19′S, 28°42′E); Mantsopa (29.1883° S, 27.0294° E); and Setsoto (28.5302° S, 27.6435° E) of the Free State Province in South Africa.

All samples were grown and cultured as described by Hoque et al. (7) and Liu et al. (8). Briefly, 9 mL of Brain Heart Infusion Broth (BHI) was inoculated with 1 mL of milk samples from the individual farms and incubated at 37°C for 24 hours. Following that, 10 µL aliquots were streaked out on mannitol salt agar (Oxoid; Thermo-Fisher, South Africa) plates and incubated for 24–48 hours at 37°C. Colony morphology on MSA was used to identify the colonies. Suspected staphylococcal colonies were sub-cultured on the nutrient agar plates and incubated at 37°C for 24–48 hours. Additionally, all pure isolate colonies were subsequently kept at −80°C in BHI broth with 15% glycerol. Quick-DNAFungal/Bacterial Miniprep Kit was used to extract genomic DNA from respective isolates according to the manufacturer’s instructions (Zymo Research, CA 92614, USA). The quantity of gDNA extracted from samples was measured using the Qubit 2.0 fluorometer (ThermoFisher, USA). Multiplexed, paired-end libraries (2 × 150 bp) were prepared using the Illumina DNA Prep kit (Illumina, San Diego, USA), followed by sequencing on the Illumina NextSeq 550 platform (Illumina, San Diego, USA) with 100× coverage at the National Institute of Communicable Diseases Sequencing Core Facility, South Africa. Analysis was conducted using the JEKESA bioinformatics pipeline v0.1; https://github.com/stanikae/jekesa (9). All underlying tools were set to default options, unless otherwise stated. Quality control and read filtering of raw paired-end reads were performed using FastQC v0.11.9, https://www.bioinformatics.babraham.ac.uk/projects/fastqc/, and TrimGalore, v0.6.2, https://github.com/FelixKrueger/TrimGalore (10) set to *Q* > 30 and length ≥ 50 bp. Species identification and closest reference detection were performed using BactInspector v0.1.3, https://gitlab.com/antunderwood/bactinspector. Contamination checks were performed using ConFindr v0.7.4 (11) and Kraken2 v 2.0.8-beta (12). *De novo* assembly was performed using SKESA v2.3.0 (13), and the assemblies were optimized using Shovill v1.1.0, https://github.com/tseemann/shovill, with depth and minimum contig length set to 100 and 200, respectively. Assembly metrics were assessed using QUAST v5.0.2, http://quast.sourceforge.net/quast (14). The assembled genomes were further investigated using the following tools: multilocus sequence typing (MLST) was performed using mlst v2.19.0, https://github.com/tseemann/mlst (—legacy—scheme saureus), based on traditional PubMLST typing schemes (https://pubmlst.org/organisms/staphylococcus-aureus/). Additionally, the assembled genome sequences were uploaded to the *S. aureus* PubMLST database (https://pubmlst.org/organisms/staphylococcus-aureus) to assign the novel sequence types.

Sequencing statistics are reported in Table 1. Since *S. aureus* is an opportunistic pathogen and a leading cause of bovine mastitis, the genome sequences produced in this study will provide a deeper understanding of the genetic variability of this pathogen in veterinary settings and will lead to a better comprehension of its pathogenic potential and improved strategies to contrast its virulence and resistance.

**TABLE 1:** Genomic characteristics of 17 *Staphylococcus aureus* strains isolated from bovine subclinical mastitis milk

Sample ID	Strain name	BioSample accession no.	GenBank accession no.	SRA accession no.	PubMLST isolate no.	MSLT	Clonal complexes	No. of raw reads	No. of contigs	Coverage depth	GC content (%)	N_50_ value (bp)	Genome length (bp)	Total no. of genes	Location
1–5	phofung01	SAMN35674906	JAUFPI000000000	SRX20883959	46374	8500	CC97	2869374	40	151	32.7	164369	2802669	2,823	Maluti a Phofung Municipality, 28°19′S, 28°42′E
1–7	phofung02	SAMN35674907	JAUFPH000000000	SRX20883960	46375	8501	CC97	3269844	33	172	32.68	145639	2781918	2,803	Maluti a Phofung Municipality, 28°19′S, 28°42′E
2–5	phofung03	SAMN35674908	JAUFPG000000000	SRX20883968	46376	8501	CC97	2612746	34	142	32.68	145649	2780593	2,801	Maluti a Phofung Municipality, 28°15′0″ S, 29° 8′ 59″ E
3–1	setsoto01	SAMN35674909	JAUFPF000000000	SRX20883969	46377	8500	CC97	2908804	42	124	32.68	122277	2799261	2,808	Setsoto Municipality 28.5302° S, 27.6435° E
3–1o	setsoto02	SAMN35674910	JAUFPE000000000	SRX20883970	46378	8500	CC97	3126264	41	157	32.69	176781	2799455	2,809	Setsoto Municipality 28.5302° S, 27.6435° E
3–4	setsoto03	SAMN35674911	JAUFPD000000000	SRX20883971	46379	8500	CC97	2755396	31	149	32.69	181801	2757166	2,771	Setsoto Municipality 28.5302° S, 27.6435° E
3–5	setsoto04	SAMN35674912	JAUFPC000000000	SRX20883972	46380	8500	CC97	2196012	32	116	32.71	341110	2788663	2,806	Setsoto Municipality 28.5302° S, 27.6435° E
3–6	setsoto05	SAMN35674913	JAUFPB000000000	SRX20883973	46381	8500	CC97	2709558	37	139	32.67	118071	2813862	2,831	Setsoto Municipality 28.5302° S, 27.6435° E
3–7	setsoto06	SAMN35674914	JAUFPA000000000	SRX20883974	46382	8500	CC97	2204122	34	116	32.69	184814	2802742	2,818	Setsoto Municipality 28.5302° S, 27.6435° E
3–9	setsoto07	SAMN35674915	JAUFOZ000000000	SRX20883975	46383	8500	CC97	2783710	32	146	32.7	156101	2756154	2,760	Setsoto Municipality, 28.5302° S, 27.6435° E
4–1o	mantsopa01	SAMN35674916	JAUFOY000000000	SRX20883961	46384	8500	CC97	2801174	31	149	32.7	210386	2802954	2,808	Mantsopa Municipality, 29.1883° S, 27.0294° E
4–4	mantsopa02	SAMN35674917	JAUFOX000000000	SRX20883962	46385	8500	CC97	1201376	56	65	32.67	106797	2813544	2,832	Mantsopa Municipality, 29.1883° S, 27.0294° E
4–7	mantsopa03	SAMN35674918	JAUFOW000000000	SRX20883963	46386	8500	CC97	2630238	39	140	32.67	225079	2815611	2,834	Mantsopa Municipality, 29.1883° S, 27.0294° E
5–1	mantsopa04	SAMN35674919	JAUFOV000000000	SRX20883964	46387	8500	CC97	1768784	38	89	32.69	181707	2802171	2,821	Mantsopa Municipality, 29.1883° S, 27.0294° E
5–1o	mantsopa05	SAMN35674920	JAUFOU000000000	SRX20883965	46388	8500	CC97	3095854	35	164	32.69	184780	2803046	2,820	Mantsopa Municipality, 29.1883° S, 27.0294° E
5–4	mantsopa06	SAMN35674921	JAUFOT000000000	SRX20883966	46389	8500	CC97	2474622	32	135	32.69	181811	2756261	2,761	Mantsopa Municipality, 29.1860° S, 27.4439° E
5–7	mantsopa07	SAMN35674922	JAUFOS000000000	SRX20883967	46390	8495	CC97	2589764	35	142	32.73	185000	2717325	2,725	Mantsopa Municipality Farm, 29.1860° S, 27.4439° E

## Data Availability

This whole-genome sequence project has been registered in DDBJ/ENA/GenBank with the BioProject accession number PRJNA981445. The Genome submission numbers, BioSample accession numbers, and PubMLST isolate IDs are provided in Table 1.
